# Regression of focal acetabular rim ossifications after periacetabular osteotomy

**DOI:** 10.1002/jeo2.70698

**Published:** 2026-03-26

**Authors:** Sufian S. Ahmad, Chiara Heller, Quentin Karisch, Marco Haertlé, Henning Windhagen, Laurentia Kort, Justus Stamp

**Affiliations:** ^1^ Department of Orthopaedic Surgery Hannover Medical School Hannover Germany

**Keywords:** developmental dysplasia of the hip, hip preservation surgery, joint instability, labral ossification, periacetabular osteotomy

## Abstract

**Purpose:**

Focal labral ossification is a recognized radiographic finding in developmental dysplasia (DDH) and is typically regarded as a chronic lesion resulting from excessive stress on the chondro‐labral junction. The necessity for direct surgical intervention at the time of periacetabular osteotomy (PAO) for these ossifications remains a subject of debate. This study aimed to explore the natural course of pre‐existing focal labral ossifications following isolated PAO in adolescents and adults, hypothesizing that biomechanical correction alone would facilitate the spontaneous resolution of this focal labral metaplasia.

**Methods:**

A retrospective observational subgroup analysis of an institutional database identified a single treatment group who underwent isolated PAO for symptomatic DDH in adolescents and adults between January 2022 and November 2024. Pre‐ and 1‐year postoperative radiographs were independently assessed for the status of the labral ossification. Radiographic parameters of acetabular coverage and validated patient‐reported outcome measures (PROMs) were compared between hips with and without ossification. Multivariable regression analysis was performed to identify factors associated with labral ossification.

**Results:**

Of 389 hips undergoing PAO, 41 (10%) demonstrated preoperative labral ossification. Baseline PROMs and most radiographic parameters were comparable between hips with and without ossification, although posterior coverage was significantly reduced in the ossification group. At 1‐year follow‐up, 37 of 41 hips (90%) showed complete radiographic resolution of labral ossification, two hips (5%) demonstrated partial regression and two hips (5%) remained unchanged. Multivariable analysis identified a mild association between posterior coverage and the presence of ossification, while no associations were found with activity level or PROMs.

**Conclusion:**

Isolated PAO was found to be strongly associated with spontaneous remission of focal rim ossification in the vast majority of dysplastic hips within 1 year after surgery. These findings challenge the concept that focal rim ossifications represent irreversible degenerative pathology and instead suggest that they are a more appropriately interpreted as a metaplastic adaptation, indicating a remodeling potential of the chondro‐labral junction following biomechanical correction alone. Routine surgical treatment of chronic labral ossifications at the time of PAO may therefore be unnecessary.

**Level of Evidence:**

Level III.

**Abbreviations**
AC: anterior coverage
AI: acetabular index
ANOVA: analysis of variance
BMI: body mass index
DDH: developmental dysplasia of the hip
EI: extrusion index
HHS: Harris Hip Score
HOOS‐PS: Hip Disability and Osteoarthritis Outcome Score–Physical Function Shortform
iHOT‐12: International Hip Outcome Tool 12‑item
LCEA: lateral center–edge angle
mHHS: modified Harris Hip Score
PAO: periacetabular osteotomy
PC: posterior coverage
PMA: Postel‐Merle d'Aubigné Score
PROMs: patient‐reported outcome measures
UCLA: University of California, Los Angeles Activity Scale
WOMAC: Western Ontario and McMaster Universities Osteoarthritis Index

## INTRODUCTION

Developmental dysplasia of the hip (DDH) is a well‐established etiology of hip pain in young adults and constitutes a primary cause of osteoarthritis [10, 12]. Chronic mechanical overload at the chondro‐labral junction in DDH frequently leads to focal rim ossification, a common radiographic finding in dysplastic hips [9, 20, 22]. Focal rim ossifications are thought to arise secondary to repetitive cycle of labral tearing and healing driven by underlying joint instability, resulting in pathological changes in the chondro‐labral junction [19, 20].

Periacetabular osteotomy (PAO) is the established operative intervention for reorienting the acetabulum to improve femoral head coverage in symptomatic, non‐arthritic adult hips and is regarded as the sole surgical procedure with the potential to alter the natural history of DDH [2, 4, 8, 18, 23]. However, there is no consensus on the optimal management of concomitant labral pathology at the time of PAO [3, 5, 6]. Some investigators advocate concurrent or antecedent labral debridement, whereas others recommend addressing the osseous deformity alone and allowing biomechanical correction to mitigate labral disease. The clinical relevance of the present study becomes particularly apparent in the context of differing surgical strategies between regions. In the United States, hip arthroscopy is frequently added to address labral pathology, whereas in Europe, PAO is more commonly performed as a stand‐alone procedure [3, 15, 16].

The present study aimed to evaluate the effect of isolated PAO on existing focal rim ossifications in dysplastic hips. Considering that adaptive and remodeling processes may vary between individuals regarding their biologics, we hypothesized that isolated PAO would be associated with complete resolution of focal rim ossifications in hips with acetabular dysplasia in response to optimization of the loading forces and stability.

## METHODS

**Figure 1:**
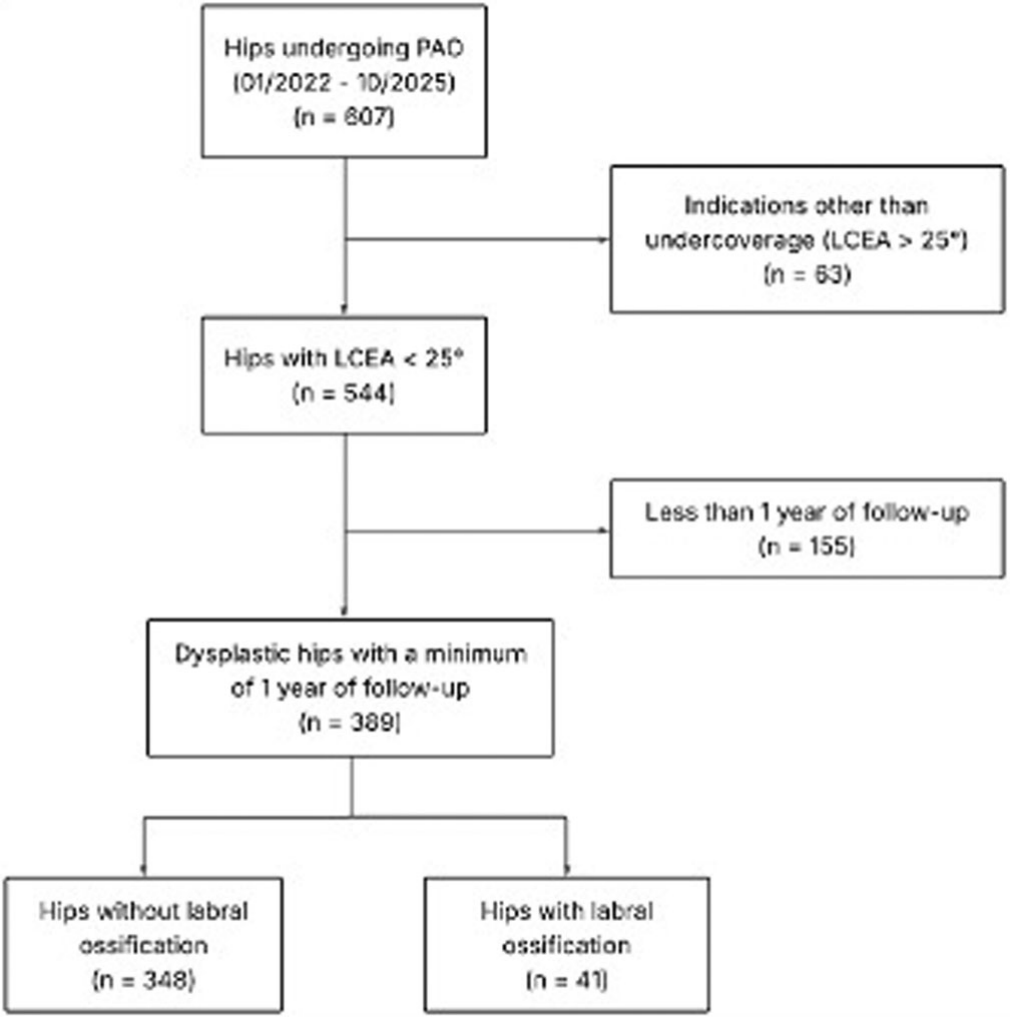
Flowchart of hips undergoing PAO. LCEA, lateral center–edge angle; PAO, periacetabular osteotomy.

An institutional PAO database was queried for this retrospective cohort study to identify patients who underwent PAO for hip dysplasia between January 2022 and November 2024. Institutional review board approval was obtained prior to study initiation, and all patients provided written informed consent. Preoperative and postoperative radiographs were reviewed for all patients who had provided written informed consent. The study protocol was approved by the institutional ethics committee. Inclusion criteria comprised adolescents and adults undergoing PAO for symptomatic hip dysplasia with a lateral center–edge angle (LCEA) < 25°. Exclusion criteria included hips treated with PAO for acetabular overcoverage or marked acetabular retroversion, as well as hips requiring concomitant procedures such as proximal femoral osteotomy, subspine correction or surgical hip dislocation. Cam‐type femoroacetabular impingement was not considered an exclusion criterion and was present in some hips. In these cases, an additional open correction of the femoral neck offset was performed via a Hueter approach. In accordance with current evidence, hips with advanced osteoarthritis were excluded, and only patients with Tönnis grade ≤ 2 were included. A total of 389 hips were included in the study cohort. The flowchart illustrating the selection process is presented in Figure 1. The mean patient age at the time of PAO surgery was 28.11 for patients without focal rim ossification and 29.56 for hips of patients with focal rim ossification. Overall, 83% of hips undergoing PAO were female, and 17% were male.

### Outcome measures

The primary outcome of this study was the presence or regression of focal acetabular rim ossification following isolated PAO. Therefore, all preoperative radiographs were evaluated by two independent investigators trained in the evaluation of plain radiographs in patients with hip pathology.

Acetabular coverage was assessed, and LCEA, anterior wall index, posterior wall index, acetabular index and extrusion index were documented.

The presence of ossification of the labrum on plain radiographs was noted as a binary measure regardless of size. All radiographs with ossification of the labrum were assessed 1 year after surgery, and the measures were documented, as well as the change in size of the ossification noted as either ‘disappeared’, ‘smaller’ or ‘unchanged’, by the two investigators. The secondary outcome of this study was whether regression of focal rim ossification was associated with a significant improvement in patient‐reported outcome measures (PROMs). To ensure reproducibility and objectivity, all radiographic images were examined by two independent experts.

As this study is a retrospective cohort analysis, no a priori sample size calculation was performed. All eligible hips undergoing PAO matching the inclusion and exclusion criteria were included in this analysis.

PROMs were obtained pre‐operatively, University of California, Los Angeles Activity Scale (UCLA), Hip Disability and Osteoarthritis Outcome Score–Physical Function Shortform (HOOS‐PS), Western Ontario and McMaster Universities Osteoarthritis Index (WOMAC), International Hip Outcome Tool 12‑item (iHOT‐12), Harris Hip Score (HHS), Modified Harris Hip Score (mHHS) and Postel‐Merle d'Aubigné (PMA) score. As PROMs are widely used and validated for assessing pain and functional outcomes in patients undergoing PAO, they were considered appropriate for this study's cohort. All PROMs were collected in patient's native language to ensure clarity and reliability.

### Result synthesis and statistical analysis

Direct observational subgroup analysis between both groups of dysplastic hips with and without radiographic ossification of the labrum was performed using analysis of variance (ANOVA). Multivariable regression analysis was performed using radiographic as well as demographic measures as input parameters and the presence of an ossification of the labrum as the output measure. Corresponding adjustments for confounding variables were performed accordingly.

## RESULTS

Patient demographics are summarized in Table 1. Of 389 hips included, 323 were female, and 66 were male. Among females, 74% showed no focal rim ossification, and 9% showed focal rim ossification. Among males, no focal rim ossifications have been observed in 15%. In 2%, focal rim ossification was apparent.

**Table 1:** Demographic data of hips undergoing PAO.

		Without ossification		With ossification	
	*n*	Mean (±SD)	*n*	Mean (±SD)	*p* value
Sex					
Female	288 (74%)		35 (9%)		
Male	60 (15%)		6 (2%)		
Additive procedures					
Femoral neck osteoplasty	52 (14%)		4 (1%)		0.400
Demographic data					
Age		28.11 (±8.16)		29.56 (±8.57)	0.219
BMI (m^2^/kg)		24.45 (±5.08)		25.35 (±5.08)	0.379
Radiographic parameters					
LCEA (°)		17.01 (±7.09)		15.16 (±8.21)	0.224
AI (°)		14.34 (±6.71)		17.05 (±8.31)	0.046
EI (%)		28.94 (±7.89)		31.53 (±9.98)	0.057
AC (%)		40.26 (±15.47)		37.31 (±17.70)	0.322
PC (%)		82.57 (±19.17)		71.30 (±21.10)	0.001
PROMS					
UCLA		5.56 (±1.94)		5.54 (±2.22)	0.976
HOOS‐PS		60.48 (±21.42)		56.07 (±25.83)	0.478
WOMAC		25.89 (±21.06)		35.13 (±26.37)	0.385
G‐FJS		20.10 (±20.94)		18.56 (±20.10)	0.131
iHOT‐12		42.35 (±23.02)		37.45 (±28.05)	0.908
HHS		71.14 (±15.45)		71.05 (±23.84)	0.715
mHHS		64.80 (±16.17)		62.53 (±19.06)	0.704
PMA		14.62 (±2.26)		14.93 (±1.71)	0.848

Abbreviations: AC, anterior coverage; AI, acetabular index; BMI, body mass index; EI, extrusion index; G‐FJS, German forgotten joint score; HHS, Harris hip score; HOOS‐PS, hip disability and osteoarthritis outcome score‐physical function shortform; iHOT‐12, International Hip Outcome Tool 12; LCEA, lateral center‐edge angle; mHHS, modified Harris hip score; PAO, periacetabular osteotomy; PC, posterior coverage; PMA, Merle d'Aubigné and postel score; PROMS, patient‐reported outcome measures; SD, standard deviation; UCLA, University of Carlifornia and Los Angeles acitvity‐level rating score; WOMAC, Western Ontario and McMaster Universities Osteoarthritis Index.

The mean age of hips without focal rim ossification was 28.11 and 29.56 in hips without focal rim ossification (*p* value 0.219). Similarly, no relevant difference was found in body mass index (BMI) with 24.45 m^2^/kg for hips without focal rim ossification and 25.35 m^2^/kg (*p* value 0.379).

Among the 389 hips that underwent PAO, 41 (10%) exhibited signs of labral ossifications. Preoperative patient‐reported outcome scores (UCLA, HOOS‐PS, WOMAC, G‐FJS, iHOT‐12, HHS, mHHS, PMA) were similar between the two groups, indicating a comparable level of symptoms and functional impairment (Table 2). Specifically, hips with focal rim ossification had a mean UCLA of 5.54 ± 2.22 and for hips without focal rim ossification of 5.56 ± 1.94 (*p* value 0.976). HOOS‐PS values averaged 56.07 ± 25.83 for ossifications and 60.48 ± 21.42 (*p* value 0.478), while WOMAC scores were 35.13 ± 26.37 for ossifications and 25.89 ± 21.06 (*p* value 0.385). No relevant difference was observed for G‐FJS with 18.56 ± 20.10 in hips with ossification and 20.10 ± 20.94 (*p* value 0.131). The same applies to the iHOT‐12. In hips with ossification, the iHOT‐12 was 37.45 ± 28.05 and 42.35 ± 23.02 in hips without ossification. HHS values were comparable between hips with ossification (71.05 ± 23.84) and hips without ossification (71.14 ± 15.45; *p* value 0.715), as were mHHS values (62.53 ± 19.06; 64.80 ± 16.17; *p* value 0.704). Pain levels assessed by the PMA also did not significantly differ between the two observed groups (14.93 ± 1.71 vs. 14.62 ± 2.26; *p* value 0.848).

**Table 2:** Radiographic data and PROMs in the 1‐year follow‐up of hips with and without labral ossifications.

	Preoperative			Postoperative		
	With ossification	Without ossification		With ossification	Without ossification	
	Mean (±SD)	Mean (±SD)	*p* value	Mean (±SD)	Mean (±SD)	*p* value
Sex						
Female	288 (74%)	35 (9%)				
Male	60 (15%)	6 (2%)				
Demographic data						
Age	28.11 (±8.16)	29.56 (±8.57)	0.219^a^			
BMI (m^2^/kg)	24.45 (±5.08)	25.35 (±5.08)	0.379^a^			
Radiographic data						
LCEA (°)	17.01 (±7.09)	15.16 (±8.21)	0.224^a^	33.48 (±6.40)	33.85 (±7.45)	0.799^a^
AI (°)	14.34 (±6.71)	17.05 (±8.31)	0.046^a^	1.85 (±4.95)	2.37 (±8.41)	0.194^a^
EI (%)	28.94 (±7.89)	31.53 (±9.98)	0.057^a^	13.07 (±0.06)	15.26 (±0.07)	0.052^a^
AC (%)	40.26 (±15.47)	37.31 (±17.70)	0.322^a^	38.77 (±0.15)	33.07 (±0.13)	0.010^a^
PC (%)	82.57 (±19.17)	71.30 (±21.10)	0.001^a^	104.55 (±1.76)	82.70 (±0.22)	0.191^a^
PROMs						
UCLA	5.56 (±1.94	5.54 (±2.22)	0.976^a^	6.72 (±1.83)	6.36 (±2.06)	0.462^a^
HOOS‐PS	60.48 (±21.42)	56.07 (±25.83)	0.478^a^	77.71 (±18.41)	75.00 (±26.24)	0.815^a^
WOMAC	25.89 (±21.06)	35.13 (±26.37)	0.385^a^	12.91 (±15.78)	19.07 (±27.99)	0.929^a^
G‐FJS	20.10 (±20.94)	18.56 (±20.10)	0.131^a^	48.43 (±29.88)	49.05 (±38.42)	0.989^a^
iHOT‐12	42.35 (±23.02)	37.45 (±28.05)	0.908^a^	62.23 (±21.91)	63.84 (±31.15)	0.438^a^
HHS	71.14 (±15.45)	71.05 (±23.84)	0.715^a^	82.62 (±17.55)	77.64 (±25.63)	0.903^a^
mHHS	64.80 (±16.17)	62.53 (±19.06)	0.704^a^	78.48 (±12.44)	74.23 (±21.47)	0.883^a^
PMA	14.62 (±2.26)	14.93 (±1.71)	0.848^a^	16.98 (±1.47)	16.00 (±2.59)	0.336^a^

Abbreviations: AC, anterior coverage; AI, acetabular index; BMI, body mass index; EI, extrusion index; G‐FJS, German forgotten joint score; HHS, Harris hip score; HOOS‐PS, hip disability and osteoarthritis outcome score‐physical function shortform; iHOT‐12, International Hip Outcome Tool 12; LCEA, lateral center‐edge angle; mHHS, modified Harris hip score; PC, posterior coverage; PMA, Merle d'Aubigné and postel score; PROMs, patient‐reported outcome measures; SD, standard deviation; UCLA, University of Carlifornia and Los Angeles acitvity‐level rating score; WOMAC, Western Ontario and McMaster Universities Osteoarthritis Index.

^a^ Mann–Whitney *U* test.

**Figure 2:**
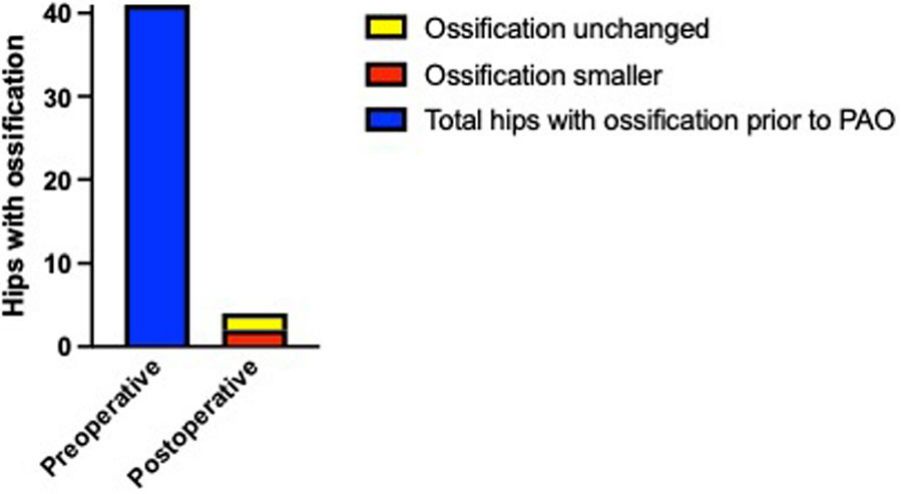
Bar chart illustrating the radiographic evolution of labral ossifications from preoperative assessment to 1‐year follow‐up after isolated periacetabular osteotomy, categorized as complete resolution, reduction in size, or no change. PAO, periacetabular osteotomy.

**Figure 3:**
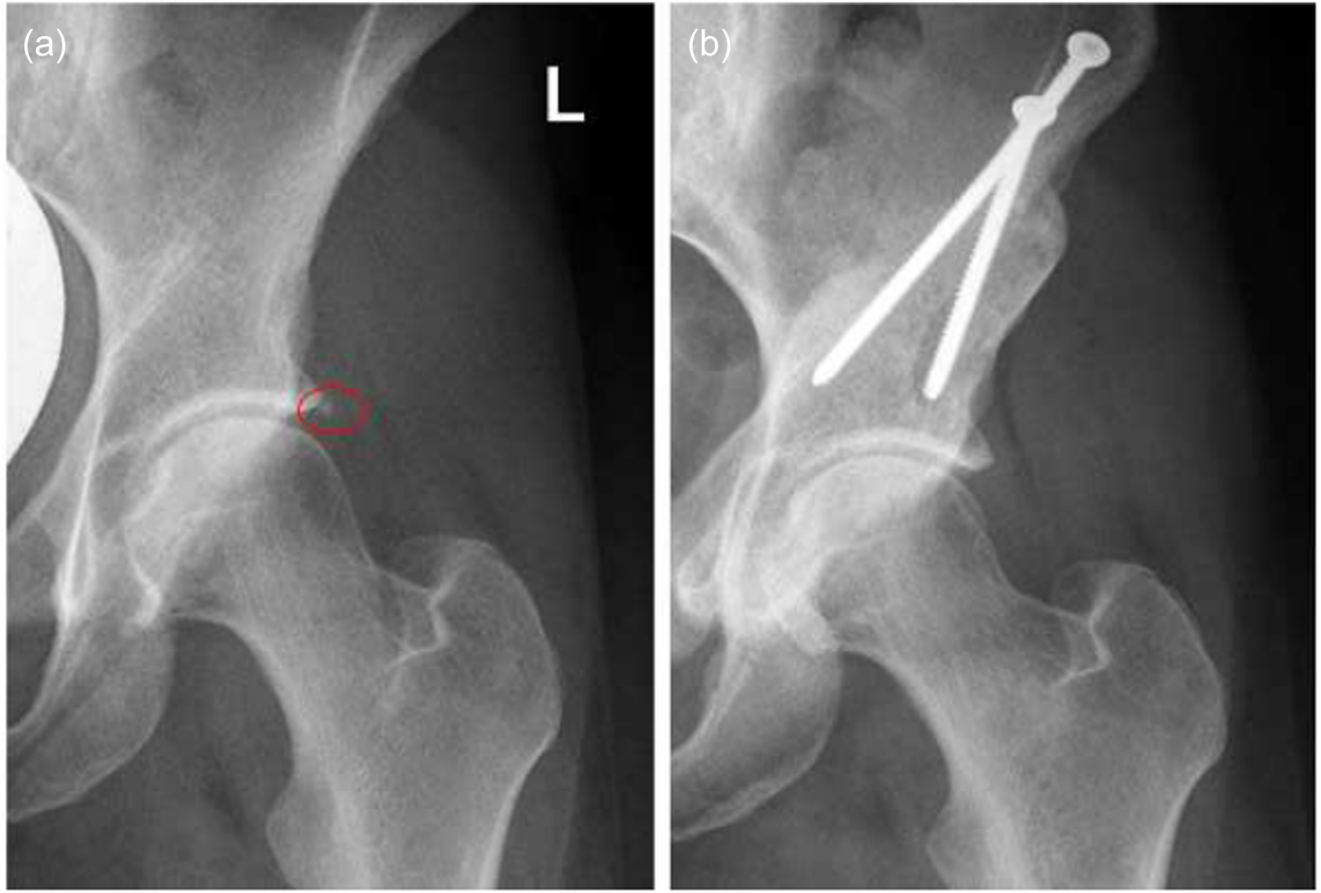
Anteroposterior pelvic radiographs of a borderline dysplastic hip showing labral ossification preoperatively (a, left hip; red circle) and complete remodellingat 1‐year follow‐up after isolated periacetabular osteotomy (b).

Of the 41 hips with labral ossifications prior to surgery, 37 (90%) demonstrated complete resolution of the ossification within the first year of follow‐up. Two hips (5%) showed a reduction in size, while 2 (5%) exhibited no change (Figure 2). Figure 3 presents an X‐ray example illustrating the resolution of labral ossification 1 year after PAO.

Multivariate analysis revealed a mild but significant association between the posterior coverage (PC) and the presence of labral ossification (Table 3). No significant associations were observed for other radiographic parameters or PROMs.

**Table 3:** Regression analysis of hips with labral ossification.

Independent factor	Beta (*β*)	Regression coefficient (*B*)	95% Confidence interval (*B*)		Sig.
Demographic data					
Age	1.041	0.040	0.990	1.094	0.120
BMI (m^2^/kg)	1.097	0.093	0.998	1.205	0.054
Radiographic parameters					
LCEA (°)	1.101	0.096	0.991	1.224	0.073
AI (°)	1.108	0.102	0.997	1.231	0.057
EI (%)	1.913	0.648	0.002	2168.707	0.857
AC (%)	0.558	−0.584	0.017	17.925	0.742
PC (%)	0.076	−2.573	0.006	0.929	0.044
PROMS					
UCLA	1.030	0.029	0.832	1.273	0.788

Abbreviations: AC, anterior coverage; AI, acetabular index; BMI, body mass index; EI, extrusion index; G‐FJS, German forgotten joint score; HHS, Harris hip score; HOOS‐PS, hip disability and osteoarthritis outcome score‐physical function shortform; iHOT‐12, International Hip Outcome Tool 12; LCEA, lateral center‐edge angle; mHHS, modified Harris hip score; PC, posterior coverage; PMA, Merle d'Aubigné and Postel score; UCLA, University of California and Los Angeles activity‐level rating score.

## DISCUSSION

The principal finding of this study was that after isolated PAO, spontaneous regression of focal rim ossification in dysplastic hips was observed within 1 year postoperatively. This observation suggests that, in the presence of focal rim ossification, additional labral surgery may not be mandatory in most cases, potentially avoiding further surgical burden or iatrogenic joint damage.

The clinical implications are supported by the observation that remodeling of the chondro‐labral junction is likely to occur following acetabular reorientation and redistribution of joint reaction forces, potentially obviating the need for additional procedures to address chronic lesions within the labrum or at the chondro‐labral junction. A finite element study by Knight et al. demonstrated that the chondro‐labral junction undergoes remodeling marked by increased cartilage contact stress and reduced labral loading [13]. In contrast to this observation, Abraham et al. demonstrated that a hypertrophied labrum remains in contact with the femur following PAO and that labral contact pressures do not necessarily normalize [1].

Given these nonuniform results, observations and outcomes from other domains of joint‐preserving surgery may further support this conceptual approach.

An analogous scenario was observed in varus malalignment of the knee with medial compartment degeneration, where corrective osteotomy has been shown to enhance the regenerative potential of the joint through unloading of the affected compartment [11, 14, 21]. While hip and knee differ substantially in anatomy and biomechanics, this study emphasizes the general concept of remodeling by redistribution of joint reaction forces. However, this comparison should be interpreted cautiously.

Interestingly, the severity of dysplasia and the level of activity did not show an association with the presence of labral ossification in the dysplastic cohort of the study. This suggests that there may be additional, less understood factors that could influence the pathological changes at the chondro‐labral junction, which require further investigation.

The clinical relevance of these findings pertains to the ongoing debate regarding concomitant procedures at the time of PAO. Labral lesions are highly prevalent in hip dysplasia, and there is no consensus on the optimal treatment strategy [9]. The disappearance of labral ossifications in most cases suggests that chronic degenerative labral lesions may not necessarily require direct surgical intervention. The likelihood that these ossifications will persist after isolated PAO is less than 10%. Clinical evidence supports this conceptual approach, indicating that PAO performed without labral intervention can achieve excellent functional outcomes and durable survivorship in patients with DDH, even in the presence of pre‐existing labral tears and chondral damage [15]. Moreover, concomitant arthroscopic labral repair did not confer a significant PROM‐based benefit over isolated PAO at follow‐up in patients with a radiologically diagnosed labral damage [7]. However, the regenerative potential is limited with respect to the complexity of labral damage. In particular, multifocal labral lesions, especially those located superiorly and extending in multiple directions, are associated with poorer postoperative PROMs following isolated PAO [17].

The main weakness of the study lies in the reliance on conventional radiography for identifying labral ossifications. While magnetic resonance imaging (MRI) may offer a more detailed assessment of the corresponding labral lesions, its application can be somewhat complex, and image quality may vary significantly. The use of conventional radiographs allows for binary statistical simplicity. Furthermore, conventional radiographs of the pelvis continue to serve as a gold standard in the radiographic evaluation of hip disease in young adults. Further limitations are based on the study design. This study is an observational subgroup analysis based on a single treatment group. Direct comparisons with untreated hips or with a matched cohort undergoing a different treatment strategy have not been performed. Consequently, causal correlations have not been depicted.

## CONCLUSION

In conclusion, most focal rim ossifications spontaneously resolve following PAO surgery without direct intervention on the labrum. This finding highlights the intrinsic remodeling potential of the chondro‐labral junction following unloading and redistribution of reaction forces. Consequently, addressing chronic labral changes during each PAO surgery may be unnecessary in most hips. Nonetheless, further research is needed to identify lesions that require treatment during long‐term follow‐up.

## AUTHOR CONTRIBUTIONS

**Sufian S. Ahmad**: Writing—original draft; formal analysis; supervision. **Chiara Heller**: Data curation; formal analysis; visualization; writing—original draft. **Quentin Karisch**: Data curation; writing—review and editing. **Marco Haertlé**: Writing—review and editing; supervision; funding acquisition. **Henning Windhagen**: Writing—review and editing; funding acquisition. **Laurentia Kort**: Data curation. **Justus Stamp**: Data curation; formal analysis; visualization; writing—review and editing.

## CONFLICT OF INTEREST STATEMENT

Sufian S. Ahmad and Henning Windhagen report grants or contracts and consulting fees from Medacta International. Henning Windhagen also discloses consulting fees Aesculap/B. Braun; payment or honoraria for lectures, presentations, speakers bureaus, manuscript writing or educational events from Aesculap/B. Braun, Medacta International and Stryker; and a leadership or fiduciary role for the Personalized Arthroplasty Society and Springer. The remaining authors declare no conflict of interest.

## ETHICS STATEMENT

The study was conducted in accordance with the Declaration of Helsinki and approved by the local Ethics Committee of the Hannover Medical School (code: 12270_BO_K_2026). Investigation was performed at Hannover Medical School, Department of Orthopaedic Surgery, Anna‐von‐Borriesstr. 1‐7, 30625 Hannover, Germany. Informed consent was obtained from all individual participants included in the study.

## Data Availability

The datasets used and analyzed during the current study are available from the corresponding author on reasonable request.
